# 1,16-Diiodo­hexa­deca­ne

**DOI:** 10.1107/S160053680800041X

**Published:** 2008-01-11

**Authors:** Naotake Nakamura, Daisuke Ishizu

**Affiliations:** a Department of Applied Chemistry, College of Science and Engineering, Ritsumeikan University, 1-1-1, Nojihigashi, Kusatsu, Shiga 525-8577, Japan

## Abstract

The mol­ecular structure of the title compound, C_16_H_32_I_2_, is centrosymmetric and the mol­ecular skeleton, including both terminal I atoms, has an all-*trans* conformation. The mol­ecules form layers of thickness *a*. These features are similar to those of the smectic C phase of liquid crystals.

## Related literature

For related literature, see: Kobayashi *et al.* (1995 ▶); Nakamura & Shimizu (2004 ▶); Nakamura *et al.* (2001 ▶); Ogawa & Nakamura (1999 ▶); Uno & Nakamura (2003 ▶).
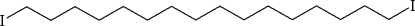


## Experimental

### 

#### Crystal data

C_16_H_32_I_2_
*M* *_r_* = 478.22
Monoclinic, 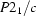

*a* = 22.0407 (11) Å
*b* = 7.4596 (13) Å
*c* = 5.7981 (18) Å
β = 96.872 (12)°
*V* = 946.5 (3) Å^3^
*Z* = 2
Cu *K*α radiation
μ = 25.96 mm^−1^
*T* = 296 (2) K
0.55 × 0.50 × 0.05 mm

#### Data collection

Rigaku AFC-5R diffractometer
Absorption correction: Gaussian (Coppens *et al.*, 1965 ▶) *T*_min_ = 0.022, *T*_max_ = 0.336
2681 measured reflections
1781 independent reflections
1560 reflections with *I* > 2σ(*I*)
*R*_int_ = 0.044
1 standard reflection every 150 reflections intensity decay: 7.5%

#### Refinement

*R*[*F* ^2^ > 2σ(*F* ^2^)] = 0.043
*wR*(*F* ^2^) = 0.137
*S* = 1.13
1781 reflections
83 parameters
H-atom parameters constrained
Δρ_max_ = 0.73 e Å^−3^
Δρ_min_ = −2.40 e Å^−3^

### 

Data collection: *MSC/AFC Diffractometer Control Software* (Molecular Structure Corporation, 1992 ▶); cell refinement: *MSC/AFC Diffractometer Control Software*; data reduction: *CrystalStructure* (Molecular Structure Corporation & Rigaku, 2001 ▶); program(s) used to solve structure: *SIR92* (Altomare *et al.*, 1994 ▶); program(s) used to refine structure: *SHELXL97* (Sheldrick, 2008 ▶); molecular graphics: *ORTEP-3 for Windows* (Farrugia, 1997 ▶); software used to prepare material for publication: *WinGX* (Farrugia, 1999 ▶).

## Supplementary Material

Crystal structure: contains datablocks global, I. DOI: 10.1107/S160053680800041X/is2269sup1.cif

Structure factors: contains datablocks I. DOI: 10.1107/S160053680800041X/is2269Isup2.hkl

Additional supplementary materials:  crystallographic information; 3D view; checkCIF report

## Figures and Tables

**Fig. 1.**
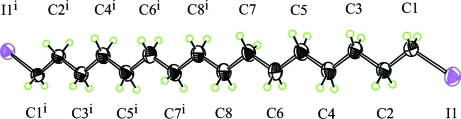
The molecular structure of (I), showing the atom numbering scheme. Displacement ellipsoids are drawn at the 50% probability level [symmetry code: (i) 1 - x, 1 - y, 2 - z].

**Fig. 2.**
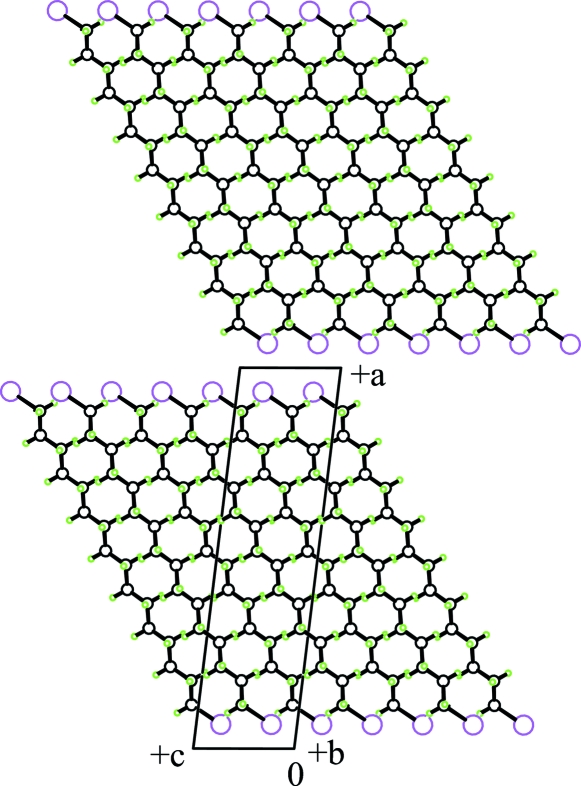
The projection of the crystal structure of (I) along the b axis.

**Fig. 3.**
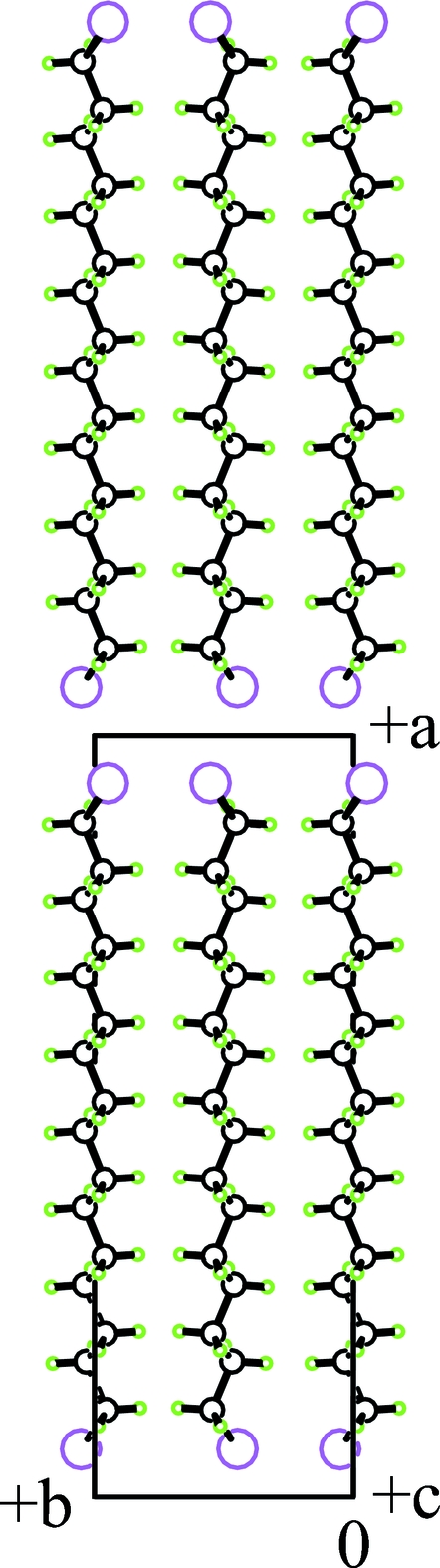
The projection of the crystal structure of (I) along the c axis.

## supplementary crystallographic information

### Comment

Normal long-chain aliphatic compounds, such are n-alkanes have been studied to elucidate the principles of a crystallization for long-chain organic compounds, because the molecular skeleton consists of a simple *trans* zigzag straight hydrocarbon chain. The molecular shape of these compounds can be regarded as a rod-like one, and the molecules in the crystalline state form a layered structure similar to those of the smectic liquid crystalline phase. Moreover, some of these long-chain compounds exhibited a high-temperature rotator phase just below their melting points, in which molecules have some degree of motional freedom, comparable with that in liquid crystals. Thus, these long-chain compounds have been studied as model compounds for smectic liquid crystals.

In order to perform the investigations of mechanism of phase transition, it is important to obtain detailed crystallographic data. Many researchers have been analyzed the crystal structure of many different kinds of normal long-chain aliphatic compounds. Recently we have systematically analyzed the crystal structures of the alkane-α,ω-diols containing 10–24 C atoms using single-crystal X-ray diffraction (Nakamura *et al.*, 2001; Uno & Nakamura, 2003), and one of the present authors has studied the phase transition phenomena of the series of the alkane-α,ω-diols containing 13–24 C atoms (Ogawa & Nakamura, 1999). In the present paper, we report a result of the crystal structure analysis of the title compound, (I), in order to clarify an effect of the terminal groups in the normal long-chain compounds on a construction of the layered structure. The molecular structure of (I) is shown in Fig. 1. The molecule is centrosymmetric and all torsion angles are close to ±180°, that is, the molecular structure including both terminal I atoms has an all-*trans* conformation. Figure 2 shows the projection of the crystal structure of (I) along the *b* axis. The molecules form layers with a thickness of *a*. In the layers, the long axes of all molecules are inclined to the *bc* plane. The layers are arranged in parallel manner between the neighboring layers, forming a bookshelf motif, as shown in Fig. 3. The molecular arrangement of (I) is similar to that of the smectic C phase of liquid crystals. In the crystal structure, the shortest I···I distance is 3.9095 (14) Å. In addition, it is attributed to the fact that the van der Waals radius of I atoms are longer than those of Cl and Br atoms, and I atoms cause strongest steric hindrance.

The results of structure analysis of 1,16-dichlorohexadecane (Nakamura & Shimizu, 2004) and 1,16-dibromohexadecane (Kobayashi *et al.*, 1995) have been reported. These compounds are arranged in a zigzag manner between adjacent layers, forming a herring-bone motif. These molecular arrangement are similar to that of the tilt smectic C phase of liquid crystals. Therefore, it is elucidated that features of the structure of (I) is differ from those of 1,16-dichlorohexadecane and 1,16-dibromohexadecane. It is considered that this difference in the crystal structure are caused by the difference of the steric hindrance of atoms located in both ends.

### Experimental

The single-crystal used for analysis was obtained by slow evaporation of a solution in a mixture of heptane and 2-propanol (1:1).

### Refinement

H atoms were positioned geometrically and treated as riding, with C—H = 0.97 Å and with *U*_iso_(H) = 1.2 *U*_eq_(C).

### Crystal data

**Table:**

C_16_H_32_I_2_	*F*_000_ = 468
*M**_r_* = 478.22	*D*_x_ = 1.678 Mg m^−^^3^
Monoclinic, *P*2_1_/*c*	Cu *K*α radiation λ = 1.54178 Å
Hall symbol: -P 2ybc	Cell parameters from 23 reflections
*a* = 22.0407 (11) Å	θ = 9.8–16.6º
*b* = 7.4596 (13) Å	µ = 25.96 mm^−^^1^
*c* = 5.7981 (18) Å	*T* = 296 (2) K
β = 96.872 (12)º	Plate, colorless
*V* = 946.5 (3) Å^3^	0.55 × 0.50 × 0.05 mm
*Z* = 2	

### Data collection

**Table:**

Rigaku AFC-5R diffractometer	θ_max_ = 70.1º
ω–2θ scans	θ_min_ = 4.0º
Absorption correction: Gaussian(Coppens *et al.*, 1965)	*h* = −26→26
*T*_min_ = 0.022, *T*_max_ = 0.336	*k* = −9→1
2681 measured reflections	*l* = −1→6
1781 independent reflections	1 standard reflections
1560 reflections with *I* > 2σ(*I*)	every 150 reflections
*R*_int_ = 0.044	intensity decay: 7.5%

### Refinement

**Table:**

Refinement on *F*^2^	H-atom parameters constrained
Least-squares matrix: full	*w* = 1/[σ^2^(*F*_o_^2^) + (0.07*P*)^2^ + 3.339*P*] where *P* = (*F*_o_^2^ + 2*F*_c_^2^)/3
*R*[*F*^2^ > 2σ(*F*^2^)] = 0.043	(Δ/σ)_max_ < 0.001
*wR*(*F*^2^) = 0.137	Δρ_max_ = 0.74 e Å^−^^3^
*S* = 1.13	Δρ_min_ = −2.40 e Å^−^^3^
1781 reflections	Extinction correction: SHELXL, Fc^*^=kFc[1+0.001xFc^2^λ^3^/sin(2θ)]^-1/4^
83 parameters	Extinction coefficient: 0.0096 (7)

### Special details

**Table:**

Geometry. All e.s.d.'s (except the e.s.d. in the dihedral angle between two l.s. planes) are estimated using the full covariance matrix. The cell e.s.d.'s are taken into account individually in the estimation of e.s.d.'s in distances, angles and torsion angles; correlations between e.s.d.'s in cell parameters are only used when they are defined by crystal symmetry. An approximate (isotropic) treatment of cell e.s.d.'s is used for estimating e.s.d.'s involving l.s. planes.

### Fractional atomic coordinates and isotropic or equivalent isotropic displacement parameters (Å^2^)

**Table:**

	*x*	*y*	*z*	*U*_iso_*/*U*_eq_	
I1	0.063509 (19)	0.44809 (7)	−0.24363 (7)	0.0532 (3)	
C1	0.1149 (3)	0.5458 (9)	0.0698 (13)	0.0473 (16)	
H1A	0.1174	0.6754	0.0628	0.057*	
H1B	0.0937	0.5146	0.2015	0.057*	
C2	0.1785 (3)	0.4687 (9)	0.1054 (12)	0.0428 (14)	
H2A	0.1995	0.4979	−0.0274	0.051*	
H2B	0.176	0.3392	0.116	0.051*	
C3	0.2148 (3)	0.5417 (10)	0.3254 (13)	0.0480 (16)	
H3A	0.193	0.5156	0.4571	0.058*	
H3B	0.2179	0.671	0.3126	0.058*	
C4	0.2789 (3)	0.4625 (10)	0.3699 (14)	0.0475 (16)	
H4A	0.2758	0.3335	0.3867	0.057*	
H4B	0.3004	0.4859	0.2366	0.057*	
C5	0.3156 (3)	0.5386 (10)	0.5854 (14)	0.0492 (17)	
H5A	0.294	0.5148	0.7185	0.059*	
H5B	0.3183	0.6676	0.5687	0.059*	
C6	0.3800 (3)	0.4614 (10)	0.6324 (14)	0.0509 (17)	
H6A	0.3772	0.3325	0.6506	0.061*	
H6B	0.4014	0.484	0.4985	0.061*	
C7	0.4170 (3)	0.5383 (10)	0.8458 (14)	0.0497 (17)	
H7A	0.3957	0.5156	0.9797	0.06*	
H7B	0.4199	0.6672	0.8276	0.06*	
C8	0.4813 (3)	0.4614 (10)	0.8924 (14)	0.0505 (17)	
H8A	0.5027	0.4843	0.7585	0.061*	
H8B	0.4785	0.3325	0.9103	0.061*	

### Atomic displacement parameters (Å^2^)

**Table:**

	*U*^11^	*U*^22^	*U*^33^	*U*^12^	*U*^13^	*U*^23^
I1	0.0459 (4)	0.0673 (4)	0.0437 (4)	−0.00104 (19)	−0.0057 (2)	−0.00042 (19)
C1	0.036 (3)	0.055 (4)	0.049 (4)	0.003 (3)	−0.004 (3)	−0.010 (3)
C2	0.036 (3)	0.053 (4)	0.037 (3)	0.005 (3)	−0.003 (3)	−0.004 (3)
C3	0.038 (3)	0.057 (4)	0.046 (4)	0.001 (3)	−0.006 (3)	−0.006 (3)
C4	0.035 (3)	0.057 (4)	0.049 (4)	0.002 (3)	−0.002 (3)	0.002 (3)
C5	0.036 (3)	0.063 (4)	0.047 (4)	0.004 (3)	−0.002 (3)	0.000 (3)
C6	0.037 (3)	0.061 (4)	0.052 (4)	0.000 (3)	−0.005 (3)	−0.003 (3)
C7	0.035 (3)	0.064 (4)	0.048 (4)	0.001 (3)	−0.003 (3)	−0.002 (3)
C8	0.037 (3)	0.061 (4)	0.051 (4)	0.003 (3)	−0.006 (3)	−0.002 (3)

### Geometric parameters (Å, °)

**Table:**

I1—C1	2.150 (7)	C5—C6	1.527 (9)
C1—C2	1.506 (9)	C5—H5A	0.97
C1—H1A	0.97	C5—H5B	0.97
C1—H1B	0.97	C6—C7	1.512 (10)
C2—C3	1.523 (9)	C6—H6A	0.97
C2—H2A	0.97	C6—H6B	0.97
C2—H2B	0.97	C7—C8	1.522 (10)
C3—C4	1.524 (9)	C7—H7A	0.97
C3—H3A	0.97	C7—H7B	0.97
C3—H3B	0.97	C8—C8^i^	1.523 (15)
C4—C5	1.515 (10)	C8—H8A	0.97
C4—H4A	0.97	C8—H8B	0.97
C4—H4B	0.97		
			
I1···I1^ii^	3.9095 (14)		
			
C2—C1—I1	112.0 (4)	C4—C5—C6	113.5 (6)
C2—C1—H1A	109.2	C4—C5—H5A	108.9
I1—C1—H1A	109.2	C6—C5—H5A	108.9
C2—C1—H1B	109.2	C4—C5—H5B	108.9
I1—C1—H1B	109.2	C6—C5—H5B	108.9
H1A—C1—H1B	107.9	H5A—C5—H5B	107.7
C1—C2—C3	111.5 (6)	C7—C6—C5	113.7 (6)
C1—C2—H2A	109.3	C7—C6—H6A	108.8
C3—C2—H2A	109.3	C5—C6—H6A	108.8
C1—C2—H2B	109.3	C7—C6—H6B	108.8
C3—C2—H2B	109.3	C5—C6—H6B	108.8
H2A—C2—H2B	108	H6A—C6—H6B	107.7
C2—C3—C4	112.8 (6)	C6—C7—C8	113.7 (6)
C2—C3—H3A	109	C6—C7—H7A	108.8
C4—C3—H3A	109	C8—C7—H7A	108.8
C2—C3—H3B	109	C6—C7—H7B	108.8
C4—C3—H3B	109	C8—C7—H7B	108.8
H3A—C3—H3B	107.8	H7A—C7—H7B	107.7
C5—C4—C3	112.7 (6)	C7—C8—C8^i^	113.8 (8)
C5—C4—H4A	109	C7—C8—H8A	108.8
C3—C4—H4A	109	C8^i^—C8—H8A	108.8
C5—C4—H4B	109	C7—C8—H8B	108.8
C3—C4—H4B	109	C8^i^—C8—H8B	108.8
H4A—C4—H4B	107.8	H8A—C8—H8B	107.7
			
I1—C1—C2—C3	178.9 (5)	C4—C5—C6—C7	179.4 (7)
C1—C2—C3—C4	178.5 (6)	C5—C6—C7—C8	−180.0 (7)
C2—C3—C4—C5	178.6 (6)	C6—C7—C8—C8^i^	−179.9 (8)
C3—C4—C5—C6	−179.7 (7)		

Symmetry codes: (i) −*x*+1, −*y*+1, −*z*+2; (ii) −*x*, −*y*+1, −*z*−1.
